# Fluorescence-Raman Dual Modal Endoscopic System for Multiplexed Molecular Diagnostics

**DOI:** 10.1038/srep09455

**Published:** 2015-03-30

**Authors:** Sinyoung Jeong, Yong-il Kim, Homan Kang, Gunsung Kim, Myeong Geun Cha, Hyejin Chang, Kyung Oh Jung, Young-Hwa Kim, Bong-Hyun Jun, Do Won Hwang, Yun-Sang Lee, Hyewon Youn, Yoon-Sik Lee, Keon Wook Kang, Dong Soo Lee, Dae Hong Jeong

**Affiliations:** 1 Department of Chemistry Education, Seoul National University, Seoul 151-744, Republic of Korea; 2 Department of Nuclear Medicine, College of Medicine, Seoul National University, Seoul 110-744, Republic of Korea; 3 Department of Molecular Medicine and Biopharmaceutical Sciences, Graduate School of Convergence Science and Technology, Seoul National University, Seoul 151-747, Republic of Korea; 4 Interdisciplinary Program in Nano-Science and Technology, Seoul National University, Seoul 151-744, Republic of Korea; 5 Laboratory of Molecular Imaging and Therapy, Cancer Research Institute, Seoul National University College of Medicine, Seoul 110-799, Republic of Korea; 6 Division of High-Risk Pathogen Research, Center for Infectious Diseases and Prevention, Korea National Institute of Health, Seoul 110-799, Republic of Korea; 7 Department of Bioscience and Biotechnology, Konkuk University, Seoul 143-701, Republic of Korea; 8 Cancer Imaging Center, Seoul National University Cancer Hospital, Cancer Research Institute, Seoul National University College of Medicine, Seoul 110-799, Republic of Korea; 9 School of Chemical and Biological Engineering, Seoul National University, Seoul 151-747, Republic of Korea

## Abstract

Optical endoscopic imaging, which was recently equipped with bioluminescence, fluorescence, and Raman scattering, allows minimally invasive real-time detection of pathologies on the surface of hollow organs. To characterize pathologic lesions in a multiplexed way, we developed a dual modal fluorescence-Raman endomicroscopic system (FRES), which used fluorescence and surface-enhanced Raman scattering nanoprobes (F-SERS dots). Real-time, *in vivo*, and multiple target detection of a specific cancer was successful, based on the fast imaging capability of fluorescence signals and the multiplex capability of simultaneously detected SERS signals using an optical fiber bundle for intraoperative endoscopic system. Human epidermal growth factor receptor 2 (HER2) and epidermal growth factor receptor (EGFR) on the breast cancer xenografts in a mouse orthotopic model were successfully detected in a multiplexed way, illustrating the potential of FRES as a molecular diagnostic instrument that enables real-time tumor characterization of receptors during routine endoscopic procedures.

There has been a growing interest in improving detection sensitivity and spatial resolution of endoscopic optical imaging in early stage cancer^1^,^2^,^3^,^4^. Meanwhile, because hemoglobin, melanin, water molecules, and other tissue components absorb and scatter light, the penetration depth of an optical signal is low, and optical imaging techniques were deemed quite challenging for *in vivo* applications. By utilizing white light reflectance, fluorescence, and chromogenic dye, the optical-based endoscopic technique visualized, in real-time, the surface morphology or even a subcellular change with high spatial resolution, allowing the discrimination of abnormal lesions from normal surfaces^5^,^6^,^7^,^8^. However, despite the advancement of endoscopic imaging capabilities, subtle neoplastic molecular changes, which are essential to determine the pathological condition, in flat and depressed lesions are difficult to identify based only on these morphological information^9^,^10^. As an alternative option, an intraoperative method was proposed to specify the tumors in such lesions so that folate-FITC was intravenously administered for targeting folate receptor-α to detect ovarian cancer^11^. Another approach was to spray fluorescent antibodies, the FITC-labeled adalimubab antibodies, over the mucosa of the colon during a colonoscopy, and it successfully visualized the mucosal membrane-bound TNF^+^ immune cells^12^. Both techniques were successfully administered in humans, and considering the difficulty in depth imaging, topical application and wide field imaging might be a preferred method.

Additional functionalities, such as Raman spectroscopy, were added to the endoscopic system to enhance the lesion detectability and to further characterize the molecular pathologies^9^,^10^,^13^,^14^,^15^,^16^. In particular, by obtaining the Raman fingerprints without any tagging agent, subtle neoplastic changes could be discerned in the pre-cancerous lesion^14^,^15^,^16^,^17^. However, intrinsic Raman endoscopy has limitations in clinical applications, such as real-time imaging, because intrinsic Raman fingerprints lack sufficient sensitivity and there are less distinctive spectral patterns between normal and cancerous lesion obliterated diagnostic accuracy due to high false-positive/negative results^18^,^19^,^20^.

To overcome these limitations, nano-probes based on surface-enhanced Raman scattering (SERS probes) were proposed as staining agents to target molecular biomarkers of the tumors^18^,^19^. The SERS probes consisted of a noble metal SERS substrate, Raman label compounds, a protective shell layer, and tumor-targeting ligands on the shell surface. These SERS probes were equipped with a unique spectral fingerprint to tag a specific bio-target with high sensitivity^21^,^22^,^23^. A strong localized surface plasmon in the vicinity of the noble metal surface dramatically enhanced the signals of the Raman label compounds by up to 10–14 orders of magnitude, and thus the Raman signals produced by SERS probes were much stronger than the intrinsic ones^24^,^25^. SERS probes have had a large multiplexing capability owing to its narrow spectral bandwidth (< 1 nm) and utilization of a single photo-excitation line for multiple targets detection^26^,^27^,^28^. Peptides, aptamers, or antibodies can be conjugated with SERS probes to identify various biomolecules by selective and specific targeting^28^,^29^.

Although SERS probes offer high sensitivity and selectivity for the molecular diagnosis of specific cancers, SERS probes-based Raman endoscopy is still limited in identifying cancerous tissues hidden within the vast normal-looking tissues. To solve this problem, a point-by-point mapping is required for the SERS probes targeted to the cancerous tissue, but scanning large areas within a practical time poses great difficulties because of the post data processing and relatively low spatial resolution^30^. Dual-modal probes integrate the fast imaging ability of fluorescence moiety and high multiplexing capability of SERS, which can be used as a clinical screening tag, in conjunction with the dual-modal imaging endoscopic techniques^31^,^32^,^33^.

This study examined the performance of a real-time *in vivo* molecular imaging of a newly developed, dual modal fluorescence-Raman endoscopic system (FRES) with fluorescence-SERS active nanoprobes (F-SERS dots) having tumor specific antibody. This dual modal FRES was developed with the following imaging and targeting strategies: (i) simultaneous detection of dual mode (fluorescence and Raman scattering) signals from dual modal nanoprobes in order to diagnose pathological lesions based on the strategy of using fluorescence images to track the target and Raman spectra to identify specifically targeted biomolecules; and (ii) utilization of optical fiber bundles, which can access *in vivo* suspicious lesions with a non-/minimally invasive procedure in conjunction with conventional endoscopy for intraoperative real-time molecular diagnostics. Fig. 1 shows a schematic overview of an *in vivo* molecular diagnostic procedure where an optical fiber bundle probe could access the vicinity of a suspicious lesion in a minimally invasive manner. To target biomolecules expressed in a cancerous tissue, the antibody-conjugated F-SERS dots were sprayed directly on the surface of the lesion. The F-SERS dots, as tumor targeting agents, were able to simultaneously emit fluorescence and SERS signals using a single excitation source. After washing the unbound F-SERS dots, the targeted area was assessed by FRES to obtain real-time fluorescence images and Raman spectra. As the fluorescence signals were produced from the F-SERS dots, the location of the target biomolecules could be tracked easily while the types of target biomolecules could be identified by the SERS signals. With this simultaneous use of an intraoperative endoscopic visualization with F-SERS dots, the feasibility of FRES could be demonstrated in a mouse orthotropic breast cancer model. FRES exhibited high sensitivity and multiplexing capability for an *in vivo* endoscopic molecular diagnosis of tumors by identifying the tumor location and characterizing the surface receptors simultaneously, which enabled rapid diagnosis of specific tumor types.

## Results

### Synthesis and characterization of the F-SERS dots

**Figure 2:**
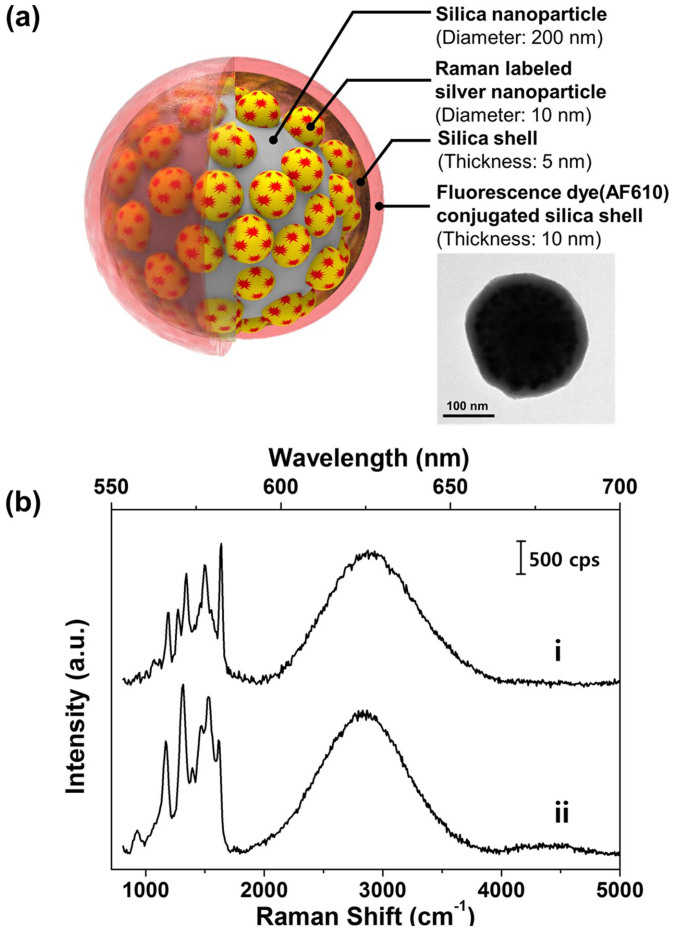
Design of the F-SERS dots. (a) A schematic composition of the F-SERS dots and the transmission electron microscopy (TEM) image of a F_AF610_-SERS_RITC_ dot (in-set). (b) Spectra of the F-SERS dots labeled with two different Raman compounds and obtained by a micro-Raman system with a 532-nm laser-line, 3.1-mW laser power, and 1-s acquisition time. Sharp spectral bands below 2000 cm^−1^ corresponded to the SERS bands of the F-SERS dots and a broad band from 2000 to 4000 cm^−1^ to the fluorescence of the AF610 dye of the F-SERS dots. Spectrum (i) was due to the F_AF610_-SERS_RITC_ dots and spectrum (ii) to the F_AF610_-SERS_FITC_ dots. The Raman bands and fluorescence band were well separated from each other and with minimal spectral overlap.

As tumor targeting agents, the fluorescence-SERS dual modal nanoprobes (F-SERS dots) were synthesized to emit fluorescence and SERS signals simultaneously using a single excitation source. As shown in Figs. 2(a) and S1, the F-SERS dots consisted of silica nanosphere as a supporting material, Raman-labeled silver nanoparticles for SERS signals, and a fluorescence dye-conjugated outer silica shell for fluorescence signals, with an overall diameter of *ca.* 240 nm (Supplementary Fig. S2). As shown in Fig. S3, an extinction band of the synthesized F-SERS dots was observed ranging broadly from 350 to 600 nm. The broadening of plasmonic extinction band can be interpreted by plasmonic couplings among silver nanoparticles on the surface of silica nanosphere. For SERS signals, the RITC and FITC compounds were utilized as Raman-label compounds. These Raman-label compounds were adsorbed on the silver nanoparticles using an isothiocyanate functional group. Additionally, AF610 was utilized as a fluorescence dye for the fluorescence signal. These F-SERS dots were further conjugated with two kinds of antibodies - anti-HER2 and anti-EGFR - for specific binding to a target, anti-HER2-F_AF610_-SERS_RITC_ dots and anti-EGFR-F_AF610_-SERS_FITC_ dots. To confirm their simultaneous emission of the fluorescence and the SERS signal, the F-SERS dots were examined on a glass slide by a micro-Raman spectrometer (LabRam 300, JY-Horiba). The distinct bands of each F-SERS dot were clearly observed (Fig. 2(b)) and well-matched with the results in the literature^34^,^35^. Furthermore, the fluorescence signal of AF610 at *ca.* 625 nm was apparent in both of the F_AF610_-SERS dots.

### Design of fluorescence-Raman endoscopic system

**Figure 3:**
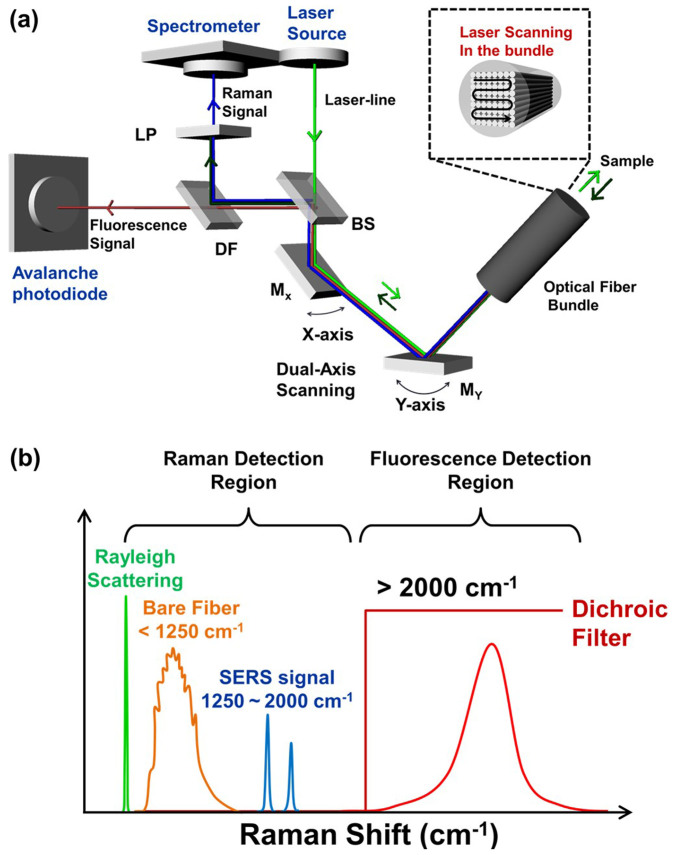
An optical design of the fluorescence-Raman endoscopic system (FRES). (a) A schematic diagram of the optical beam path. M_X_: Oscillating mirror for X-axis, M_Y_: Oscillating mirror for Y-axis, BS: Beam splitter, DF: Dichroic filter, and LP: Long pass Raman edge filter. (b) An illustration of the spectral design sectioning the ranges of the collected lights from the sample. The collected lights contained four different kinds of signals: The scattered laser-line (Rayleigh scattering), the Raman scattering signal intrinsic to the optical fiber, and the fluorescence and the SERS signals from the F-SERS dots.

The fluorescence-Raman endoscopic system (FRES) simultaneously detected both fluorescence and Raman signals in real-time, capitalizing on an intense fluorescence signal for an acute detection of the target location and on a narrow bandwidth of the Raman scattering for multiplexed analysis. In *in vivo* dual-modal endoscopic detection, the FRES consisted of three components, as shown in Fig. 3(a): i) a dual-axis laser-scanning unit to allow for light incidence to and light collection from an entire optical fiber bundle; ii) a separation unit for fluorescence and SERS signals to simultaneously detect both signals from the targeted F-SERS dots; and iii) two detection units that consisted of an avalanche photodiode for fluorescence imaging and a spectrometer equipped with a CCD camera for SERS spectral measurements. As an excitation source, a 532 nm laser was utilized to generate SERS-effective Raman signals of the F-SERS dots and simultaneously excite the fluorescence dyes of the F-SERS dots. The spectral window was divided into two sections (Fig. 3(b)): One for the fluorescence signals above 2000 cm^−1^ from a 532 nm laser-line, and the other for the Raman scattering signals from 1250 to 2000 cm^−1^ to minimize an optical noise from the optical fiber. The paths of the optical beams from the fluorescence and Raman signals were completely separated and independently detected, and thus the FRES enabled simultaneous, real-time detection of the fluorescence images and Raman spectra with a single laser-line excitation (Supplementary Fig. S4 and Video. S1).

Signals were collected through a dual-axis laser-scanning unit that was composed of two oscillating mirrors for real-time imaging. The excitation laser lights sequentially illuminated into *ca.* 100,000 individual optical fibers in the entire fiber bundle optical guide through two orthogonally oscillating mirrors at 4 kHz. The signals were collected from the individual optical fibers using an identical optical pathway as the excitation light, and they were then separated into the fluorescence and the Raman signal by a dichroic filter. At this stage, each point from the individual optical fibers collecting fluorescence signals were synchronized with each pixel of the processed fluorescence image. This allowed the FRES to rapidly construct the fluorescence images (12 frames/s) that enabled real-time tracking of the tumor-targeting F-SERS dots in a large area (Supplementary Fig. S5). For simultaneous, dual-modal detection, a fluorescence dye was used for the F-SERS dots, which have a maximum emission wavelength over 593 nm when the fluorescence dye was excited by a 532 nm laser-line. AF610 was chosen among four dye candidates - AF568, AF594, AF610, and AF633 - since it showed the lowest background noise in the Raman detection range and sufficiently intense fluorescence signals to construct images in the fluorescence detection range (Supplementary Fig. S6).

The reflected light from a fluorescence-passing filter (below 2000 cm^−1^ from the laser-line) contained Rayleigh and Raman scattering light. The light was then passed through an edge filter to block Rayleigh scattering before entering a spectrometer equipped with a CCD detector for Raman detection (Supplementary Fig. S4(b)). As the strong intrinsic Raman signals below 1250 cm^−1^ were produced by optical fibers, a spectral window with a low background noise was selected, ranging from 1250 to 2000 cm^−1^ (Supplementary Fig. S7). For Raman-label compounds in SERS detection, the RITC and FITC dye molecules were used since they have strong and distinct bands without any spectral overlap in the SERS detection range.

### Evaluation of the fluorescence-Raman endoscopic system (FRES)

**Figure 4:**
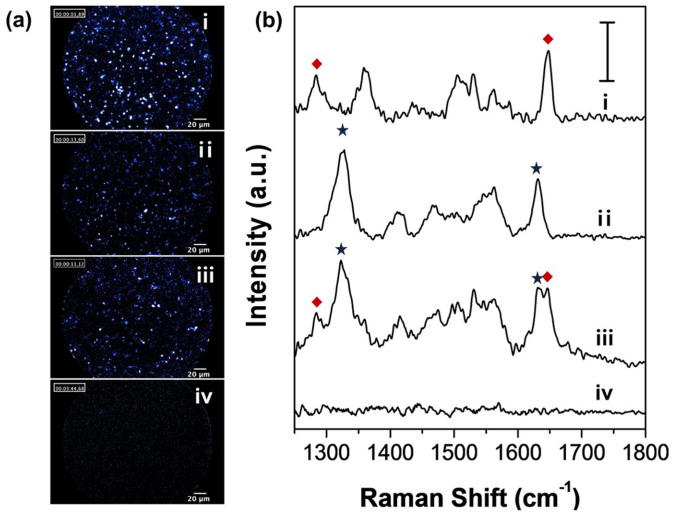
Fluorescence images (a) and Raman spectra (b) of the two different F-SERS dots, their mixture, and phosphate buffer solution (PBS) as a blank reference. Sample (i) is the F_AF610_-SERS_RITC_ dots (♦), sample (ii) is the F_AF610_-SERS_FITC_ dots (★), sample (iii) is a mixture of the two F-SERS dots, and sample (iv) is the PBS in a conical tube. All fluorescence images and Raman spectra were obtained using the FRES instrument with a laser power of 2.7 mW at the sample. All fluorescence images were represented by a false color. The Raman spectra were integrated for 1 s, and the scale bar in the spectra represents 100 counts per second.

To evaluate the ability of the dual modality detection of the F_AF610_-SERS_RITC_ and F_AF610_-SERS_FITC_ dots, each F-SERS dots and their mixture in PBS were prepared separately in a conical tube, and their fluorescence and Raman signals were measured simultaneously by FRES. Fig. 4 shows fluorescence images and Raman spectra simultaneously obtained by FRES. The fluorescence images of each kind of F-SERS dots and their mixture exhibited bright small dots were clearly distinguished from a background image (Fig. 4(a)). In the Raman spectra, distinct bands of F-SERS dots could be identified from the mixture along with those from the individual F-SERS dots: 1285 and 1648 cm^−1^ bands for the RITC coded F-SERS dots; and 1324 and 1633 cm^−1^ bands for the FITC coded F-SERS dots (Fig. 4(b)). In contrast, the fluorescence images and Raman spectra did not show any signals from the F-SERS dots in PBS.

**Figure 5:**
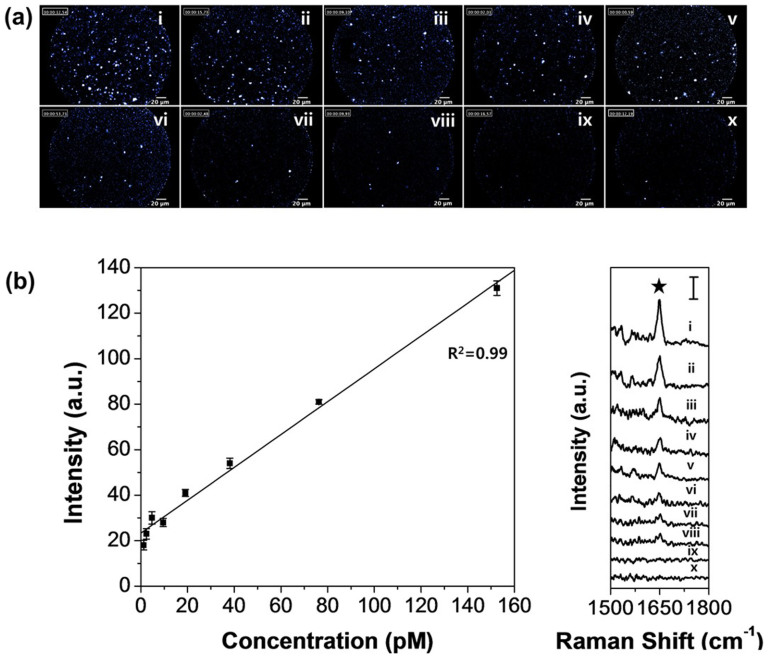
Evaluation of the detection limit of fluorescence and SERS signal using the FRES. (a) Fluorescence images of different concentrations of the F_AF610_-SERS_RITC_ dots: From (i) to (x), the concentration is 152, 76, 38, 19, 10, 5, 2, 1, 0.5, and 0.3 pM, respectively. (b) The left side plot shows the SERS intensity profile of the 1648-cm^−1^ band of the RITC Raman compound (★). The right side plot shows the Raman spectra of the F_AF610_-SERS_RITC_ dots in different concentrations. All the fluorescence images were represented by a false color. All Raman spectra were measured at a laser power of 2.7 mW and an acquisition time of 1 s. The Raman spectra were obtained with 2.7-mW laser power and 1-s integration. The scale bar in the spectrum represents 50 counts per second.

The intensities of the fluorescence and Raman scattering of the F_AF610_-SERS_RITC_ dots in different concentrations (152 to 0.3 pM in PBS) were measured in a conical tube by FRES to determine their sensitivity. Fluorescence images and Raman spectra were obtained from each concentration of the F_AF610_-SERS_RITC_ dots at a laser power of 2.7 mW. The fluorescence images in Fig. 5(a) show that the number of bright dots decreased with a decreasing concentration of the F_AF610_-SERS_RITC_ dots. In the Raman spectra (Fig. 5(b)), the SERS intensity of the F_AF610_-SERS_RITC_ dot peak at 1648 cm^−1^ also decreased linearly as the concentration of the probe decreased (*R*^2^ = 0.99). The minimal detectable concentration was approximately 1 pM for the F_AF610_-SERS_RITC_ dots with an optical fiber bundle probe possessing a 240-μm field of view and a 10-μm optical sectioning.

### *In vivo* and intraoperative endomicroscopic detection of targets on the tumor

**Figure 6:**
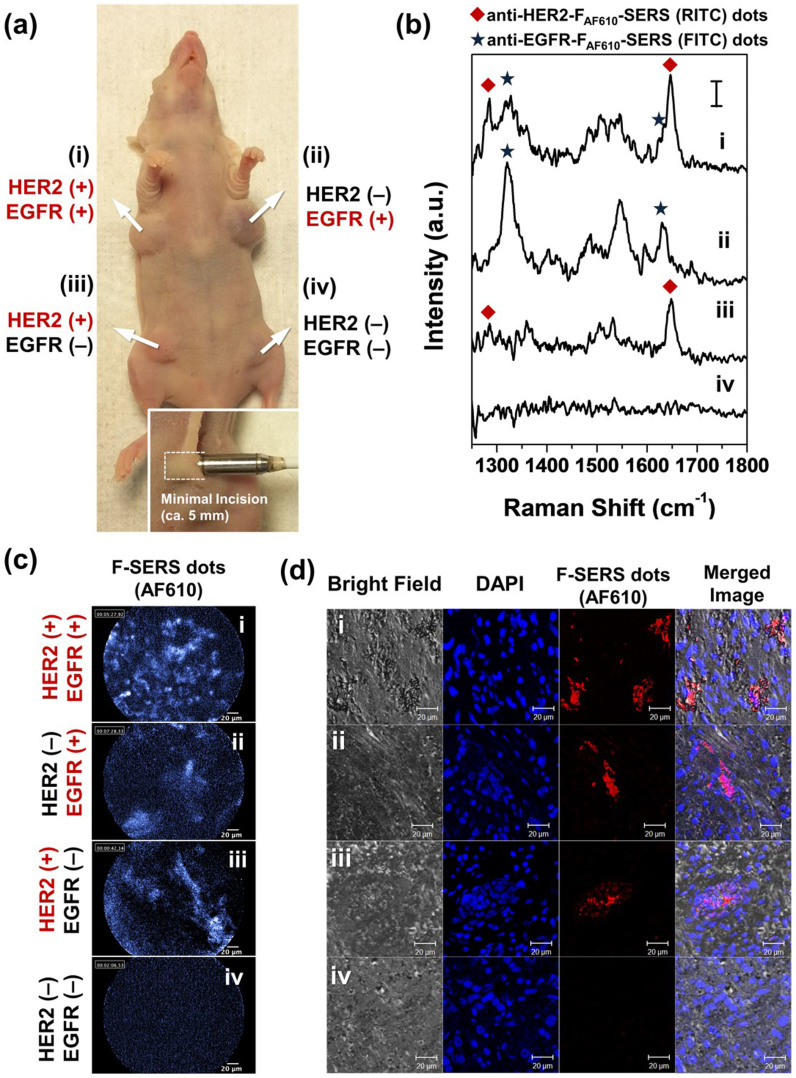
Demonstration of an *in vivo* active targeting ability of the F-SERS dots on the HER2 and EGFR positive breast tumor xenografts. Each tumor site (i) was not treated with any antibody, (ii) pre-treated with the anti-HER2 antibody, (iii) pre-treated with the anti-EGFR antibody, or (iv) pre-treated with both antibodies for blocking specific binding. Then, they were treated with the F-SERS dots (anti-HER2-F_AF610_-SERS_RITC_ dots and anti-EGFR-F_AF610_-SERS_FITC_ dots) for the FRES imaging. (a) A photograph of the tumor-bearing mouse with the receptor expression status as shown besides the image. The real-time fluorescence images and Raman spectra were simultaneously obtained with an optical fiber bundle probe of the FRES (lower box). (b) Fluorescence images were obtained by the FRES with real-time (12 frames/s). The bright area in fluorescence images corresponds to the targeted probes. (c) The Raman spectra were obtained by the FRES at a laser power of 2.7 mW and acquisition time of 1 s. The observed Raman bands in the Raman spectra correspond to the RITC (♦) and FITC (★) from the F-SERS dots. (d) The confocal fluorescence laser scanning (CLSM) images of the tumor sites. The nuclei of the tumor cells stained with 4’,6-diamidino-2-phenylindole (DAPI) dye were shown as blue spots, and the targeted F-SERS dots containing Alexa Fluor 610 were shown as red spots.

To confirm the feasibility of an endoscopic molecular diagnostics with FRES from the perspective of an intraoperative examination, four xenograft model mice were produced from the MDA-MB-231/HER2 breast cancer cell line, which expressed high levels of HER2 and EGFR (Supplementary Fig. S8). The active targeting was performed on the tumor surface (i.e., outer surface) and on the cut surface of the tumor 5-mm deep from the outer surface, respectively. Since subcutaneous breast cancer implants in mice were used, we first incised the skin of the breast near the tumor and simulated endoscopic imaging by probing the outer surface of the tumors and subsequently of their cut surface. After pretreatment with blocking antibodies, the targeting F-SERS dots (the anti-HER2-F_AF610_-SERS_RITC_ dots and anti-EGFR-F_AF610_-SERS_FITC_ dots) were sprayed directly on the surface of the tumor tissue. After incubation for 10 min, the unbound F-SERS dots were washed three times with PBS (pH 7.4). The Raman bands and fluorescence were simultaneously detected by a minimally invasive injection of the optical fiber bundle probe of the FRES (Fig. 6(a)).

Fig. 6 shows a representative result from the four sets of the tumor surface studies. For the tumor site (i) in Fig. 6(b), the distinct SERS bands in the Raman spectra were observed at 1285 and 1648 cm^−1^ corresponding to the anti-HER2-F_AF610_-SERS_RITC_ dots, and at 1324 and 1633 cm^−1^ corresponding to the anti-EGFR-F_AF610_-SERS_FITC_ dots. At the same time, the location of the targeted F-SERS dots could be identified as a bright area in a dark background of the non-targeted lesions ((i) in Fig. 6(c)), thereby indicating that the intense fluorescence signals of these F-SERS-dots with the AF610 dye can rapidly track the targeted regions. For the tumor site (ii) in Fig. 6(b), which was treated with an anti-HER2 antibody to block the HER2 receptor, distinct SERS bands at 1324 and 1633 cm^−1^ corresponding to the anti-EGFR-F_AF610_-SERS_FITC_ dots were observed, without the bands of the anti-HER2-F_AF610_-SERS_RITC_ dots at 1285 and 1648 cm^−1^. A bright area was observed in the fluorescence images ((ii) in Fig. 6(c)). In contrast, at the anti-EGFR antibody-treated tumor site (iii) in Fig. 6(b), the distinct bands at 1285 and 1648 cm^−1^ corresponding to the anti-HER2-F_AF610_-SERS_RITC_ dots were only observed in the Raman spectra. The targeted locations also appeared as a bright area in the fluorescence images ((iii) in Fig. 6(c)). In the case of both antibodies-treated tumor site (iv) in Fig. 6(b) and 6(c), we did not observe either fluorescence or SERS signals. As shown in Fig. 6(b) and 6(d), only the targeted tumor areas exhibited fluorescence signals from the F_AF610_-SERS_RITC/FITC_ dots (represented by blue color in the FRES images or red color in the confocal laser scanning microscopy images), thus indicating that the antibody-conjugated F-SERS dots were specifically bound to the HER2 and EGFR expressing tumor tissues. To validate reproducibility of FRES in an endoscopic multiplexed molecular diagnosis, the entire results of the multiplex detection in the outer and cut surfaces of the tumor in the four mice were categorized into three grades: ‘clearly detectable’, ‘just-noticeably detectable’, and ‘non-detectable’. For the outer surface study, 75% (3/4) was graded as ‘clearly detectable’, and 25% (1/4) was graded as ‘just-noticeably detectable’. For the cut surface study, 100% (4/4) of tumors were graded as ‘clearly detectable’. Non-detectable cases were not found in either surface of tumor (Supplementary Table S1 and Fig. S9).

## Discussion

In this study, a novel endoscopic system using fluorescence-SERS active nano-probes has demonstrated successfully an *in vivo* dual modal detection of multiple targets for specific cancer tissue. The technique was based on simultaneous detection of dual modalities involving fluorescence and Raman signals using a single laser-line of excitation. This approach utilized F-SERS dots as tumor-targeting agents by directly spraying them on a suspicious lesion, thereby allowing for a real-time endoscopic multiplexed molecular diagnostics. Compared to previous fluorescence and/or Raman endoscopic diagnosis techniques^18^,^19^,^36^,^37^, our strategy of using the FRES with F-SERS dots showed great potential for clinical diagnostics. For example, although fluorescence endoscopy provides highly sensitive fluorescence images, it does not have multiplexing capabilities due to its broad bandwidth of fluorescence emission. In contrast, Raman endoscopy with SERS dots as targeting agents can simultaneously detect subtle molecular changes and multiple bio-targets. However, the Raman endoscopy is limited for a clinical use because it can characterize only a small portion of an extensive suspicious lesion. To address these limitations, we have optimized the spectral design and endoscopic instrument. The F-SERS dots were fabricated to emit fluorescence and SERS signals at the same time by a single excitation source, and the FRES was designed to simultaneously detect fluorescence and Raman signals as a real-time imaging instrument. Although the FRES cannot provide a wide-field endoscopic imaging (i.e., over several centimeters)^11^,^36^, the real-time fluorescence imaging ability (240 μm field of view, 12 frames/sec) of the FRES allowed for rapid localization of a pathologic lesion in the extensively suspicious area of interest^37^,^38^, while the Raman spectra enabled the identification of different types of F-SERS dots bound to multiple bio-targets. Furthermore, the FRES utilized the optical fiber bundle with an external diameter of *ca.* 2.6 mm, which can be inserted into the instrument channel of a conventional endoscope. This feature could enable the FRES to be intraoperatively utilized for molecular diagnosis during a well-established endoscopic examination without further modification of the conventional endoscopic instrument.

The FRES exhibited high sensitivity to detect a low concentration of F-SERS dots as tumor targeting agents (as low as 1-pM). This detection limit showed much greater sensitivity than that of other previously reported Raman-based fiber optic devices that relied only on intrinsic Raman scattering^9^,^15^,^16^. This feature met the sensitivity standard to be used as a clinical diagnostic tool^10^,^19^. However, although small molecules like folate could evade reticuloendothelial system after intravenous administration^11^, the nanoparticle-based targeting agents accumulated in the liver and spleen by a mononuclear phagocytic system in an intravenous injection of nanoparticles for *in vivo* targeting^18^,^39^,^40^,^41^,^42^. By utilizing the direct topical administration of the tumor-targeting probe, the FRES was able to circumvent major limitations from the accumulation of nanoparticles, such as potential toxicity and low targeting efficiency. Since topically administered targeting agents can reach and bind to their targets more effectively than those from intravenous injection, the administration method used in FRES potentially increases the targeting efficiency^10^,^12^.

In this study, the reproducibility and applicability of FRES for the *in vivo* multiplex molecular diagnostics were demonstrated by direct topical administration of the antibody conjugated-F-SERS dots on the outer and cut surfaces of the breast tumor xenografts in the four mice. The antibody conjugated-F-SERS dots selectively targeted the corresponding the HER2 and EGFR on the outer and cut surfaces of the cancerous tissues. In terms of a multiplexed molecular diagnostics, the HER2 and EGFR were identified clearly at the same time on the outer and cut tumor surfaces in the four mice with 75% and 100% accuracy, respectively. For only one case in the outer surface, two kinds of biotargets were just-noticeably detectable without any significant error. The observed small variation was understood probably due to the variation of the HER2/EGFR expression according to the size and physiological conditions of the tumor implants. These results demonstrated that the real-time fluorescence imaging and Raman spectral measurement provided easy identification of the location of the culprit lesion and the types of targeted biomolecules thereof in an extensive area of suspected cancerous tissues.

When compared to immunohistochemistry analysis which has shortcomings such as reduced time efficiency, high background stain, and antigen transformation by solvents, the multiplex F-SERS dot approach using the topical spray can detect tumor biomarkers in the bona fide tissue environment without any endoscopical manipulation or without major skin removal in case of intraoperative imaging. In addition, the FRES system has the capability of simultaneous SERS multiplexing accompanied by the fast-screening search by fluorescence signals, whereas confocal laser endomicroscopy is limited in multiplexing capability due to its broad bandwidth of fluorescence emission. Since the F-SERS dot strategy aimed to provide characteristics of the tumor on site by simultaneous monitoring of several tumor antigens in the primary tumor, it could contribute to an earlier detection of breast cancer in a patient with certain biomarkers that have therapeutic or prognostic significances^12^, and could help design patient-tailored therapeutic drugs for customized and personalized cancer therapy.

Some limitations existed in our study in order to directly translate the FRES to clinical use. Although the silica-encapsulated F-SERS dots showed non-toxicity in a cell viability assay in a previously reported study^33^, further studies investigating the long-term toxicity of the F-SERS dots need to be performed. Furthermore, a minimum working dose of F-SERS dots for topical administration needs to be identified to minimize the potential toxicity from targeted F-SERS dots after examination. Additionally, to truly discriminate between overexpression and normal expression of receptors, further studies for negative targeting on the healthy breast tissue need to be conducted.

In conclusion, we have demonstrated a sensitive and efficient fluorescence-Raman endoscopic system for *in vivo* multiplexed molecular diagnostics, highlighting its sensitivity, real-time dual modal detecting ability, and multiplexed active targeting. In breast cancer xenografts, the HER2 and EGFR expressed in the tumor tissues were identified using antibody conjugated F-SERS dots by the FRES in a minimally invasive procedure. As illustrated in our results, the real-time endoscopic molecular imaging technique using fluorescence and Raman dual-modality could potentially be applied to a routine endoscopic procedure where multiplexed molecular diagnosis of specific cancers or the differentiation of precancerous tissue from normal tissue in the early stage is critical. In addition, this unique detection strategy can be applied for an accurate and efficient intraoperative molecular diagnosis of all tissues of the hollow organs accessible to an endoscope.

## Methods

### Chemicals and materials

Tetraethyl orthosilicate (TEOS), 3-mercaptopropyltrimethoxysilane (MPTS), ethylene glycol (EG), silver nitrate (AgNO_3_, > 99.99%), 3-aminopropyltriethoxysilane (APTES), octylamine (OA), rhodamine B isothiocyanate (RITC), fluorescein isothiocyanate (FITC), dimethyl sulfoxide (DMSO), *N*-methyl-2-pyrrolidone (NMP), *N*-hydroxysuccinimide (NHS), *N,N′*-diisopropylcarbodiimide (DIC), *N,N′*-diisopropylethylamine (DIPEA), 4-dimethylaminopyridine (DMAP), and bovine serum albumin (BSA, > 98%) were purchased from Sigma-Aldrich Inc. (St. Louis, MO, USA) and used without further purification. Absolute ethanol (99.9%), ammonium hydroxide (NH_4_OH, 27%), 2-propanol (99%), and ethanol (98%) were purchased from Daejung Chemicals (Siheung, Korea). Succinimidyl esters conjugated with Alexa Fluor (AF) 563, 594, 610-X, and 633 were purchased from Invitrogen Inc. (Carlsbad, CA, USA). Phosphate-buffered saline (PBS; 137 mM NaCl, 10 mM Na_2_HPO_4_, 2.0 mM KH_2_PO_4_, and 2.7 mM KCl at pH 7.4) was prepared in-house. Deionized (DI) water was used for all experiments. Herceptin (anti-human epidermal growth factor receptor 2 (anti-HER2) monoclonal antibody) and Cetuximab (anti-epidermal growth factor receptor (anti-EGFR) monoclonal antibody) were purchased from Merck Millipore (Darmstadt, Germany).

### Fluorescence-Raman endoscopic system (FRES)

The FRES used a continuous wave diode-pumped solid-state 532-nm laser (Cobolt Samba^TM^, Cobolt, Sweden) coupled with a single mode fiber for light delivery. A custom-made dual-axis laser scanning unit (Cell-Vizio, Mauna Kea Technologies, France) combined with a spectroscopic system for real-time fluorescence imaging and multiplexed Raman detection utilized an optical fiber bundle for delivery of the incident light to the sample and collection of the lights from it^43^. This optical fiber bundle consisted of *ca*. 100,000 optical fibers of 2-μm diameter and had an overall external diameter of 2.6 mm. The working distance of the optical fiber bundle was 60 μm and its field of view was 240 μm. The separation unit used two optical filters: A long-pass dichroic filter (FF593-Di03-25D, Semrock Inc., Rochester, NY) for separation of scattering light and fluorescence light and an edge filter (LP03-532RS-25, Semrock Inc.) for separation of Rayleigh- and Raman-scattering light. To detect Raman signals, a spectrometer (SR 303i-A, Andor Technology, Belfast, UK) with a thermo-electrically cooled CCD detector (DV401A-BV, Andor Technology) was utilized. The fluorescence signal was collected by an avalanche photodiode and processed into real-time images by the imaging software (ImageCell, Mauna Kea Technologies, France).

### Preparation of fluorescence surface-enhanced Raman scattering probes (F-SERS dots)

The *ca.* 200-nm sized silica nanoparticles (NPs) were synthesized by the Stöber method^44^. Tetraethyl orthosilicate (TEOS, 1.6 mL) was dissolved in 40 mL of absolute ethanol, followed by addition of ammonium hydroxide (NH_4_OH, 27%, 3 mL). The resulting mixture was vigorously stirred using a magnetic bar for 20 h at 25°C. The silica NPs were centrifuged and then washed with ethanol several times to remove excess reagents. These silica NPs were then functionalized with thiol groups as described below. The silica NPs (100 mg) were dispersed in 2 mL of ethanol containing 100 μL MPTS and 20 μL NH_4_OH (27%). The mixture was stirred for 2 h at 25°C. Subsequently, the resulting thiol-functionalized silica NPs were centrifuged and washed with ethanol several times. One hundred milligrams of thiol-functionalized silica NPs was thoroughly dispersed in 50 mL of AgNO_3_ solution (3 mM in ethylene glycol). Octylamine (82.7 μL, 10 mM) was then rapidly added to the above dispersion and stirred for 1 h at 25°C. Octylamine was used as both a reducing agent and as a stabilizer for the mild nucleation of the silver NPs on the surface of the silica NPs. The resulting silver-embedded silica (Ag Si) NPs were then centrifuged and washed with ethanol several times for purification. These Ag Si NPs were labeled with two kinds of Raman-label compounds (RITC and FITC). Ten milligrams of Ag Si NPs were dispersed in a 1 mL ethanol solution containing 1 mM of each Raman label compound. The dispersion was shaken for 30 minutes at 25°C. The Raman-labeled Ag Si NPs (SERS NPs) were centrifuged and washed with ethanol two times to remove excess reagents. These SERS NPs were coated with a silica shell to prevent aggregation and fluorescence quenching as described below^31^. Ten milligrams of SERS NPs was dispersed in 24 mL of a solution containing 2-propanol (20 mL) and water (4 mL). Then, NH_4_OH (27%, 600 μL) was then added to this dispersion. Next, TEOS (7 μL) was added into the mixed dispersion three times each at the intervals of 1 h and then stirred for 24 h. The silica-coated SERS NPs were centrifuged and washed with ethanol several times to remove excess reagents. Next, these silica-coated SERS NPs were encapsulated by a fluorescence dye conjugated to the silica shell. A 50 μL of the APTES (19.2 mM in ethanol) and 5 μL of the AF610 (8 mM in DMSO) were mixed to allow the conjugation of the APTES and fluorescence dye. The resulting solution was stirred for 15 h at 25°C. A 10 mg portion of silica-coated SERS NPs were dispersed in 24 mL of a mixed solution containing 2-propanol (20 mL) and water (4 mL). Then, 55 μL of the AF610-APTES conjugated ethanol solution was added to this dispersion. Subsequently, 10 μL of the TEOS and 0.5 mL of NH_4_OH (27%) were added. The dispersion was stirred for 9 h at 25°C. The final nanoprobes (the F_AF610_-SERS_RITC/FITC_ dots) were centrifuged and washed with ethanol several times for purification.

### Immobilization of antibodies on F-SERS dots

A 1 mg portion of the F-SERS dots was dispersed in 1 mL of the APTES solution (5 vol% in ethanol), and 10 μL of NH_4_OH (27%) was added. The resulting dispersion was vigorously stirred for 1 h at room temperature. The APTES-treated F-SERS dots were washed with ethanol several times, and then re-dispersed in 500 μL of NMP. Succinic anhydride (1.75 mg) was added to the APTES-treated F-SERS dots dispersion, followed by adding 3 μL of DIPEA to introduce carboxyl groups on the F-SERS dots. The resulting mixture was stirred for 2 h. Subsequently, the carboxyl group-functionalized F-SERS dots were washed with DMF three times and then re-dispersed in 200 μL of anhydrous DMF. Then, DIC (2.7 μL), NHS (2 mg), and DMAP (0.21 mg) were added to the dispersion of carboxyl group-functionalized F-SERS dots to activate the carboxyl groups for antibody conjugation. The resulting mixture was stirred for 2 h and then washed with PBS (pH 7.4) three times. Herceptin (50 μg, Trastuzumab; anti-HER2/neu receptor monoclonal antibody) or Cetuximab (50 μg; anti-EGFR monoclonal antibody) was added to the NHS-activated F_AF610_-SERS_RITC/FITC_ dots dispersed in 200 μL of PBS. The mixture was incubated for 1 h at 25°C. The antibody-immobilized F-SERS dots were centrifuged and washed with PBS containing 0.1% (w/v) Tween 20 and PBS, consecutively. Finally, the antibody-immobilized F-SERS dots were treated with BSA (1% (w/v) in PBS solution, pH 7.4) for 30 minutes and washed with PBS solution containing Tween 20 and PBS.

### *In vivo* orthotopic breast cancer model

All animal studies were conducted according to the protocols approved by the Institutional Animal Care and Use Committee of the Seoul National University. The human breast cancer cell line MDA-MB-231/HER2, a HER2-expressing variant of MDA-MB-231 (ATCC, Manassas, VA) was used for this research. Cancer cells were cultured with Dulbecco's modified Eagle's medium (DMEM; Welgene, Daegu, Korea) containing 10% Fetal Bovine Serum and 1% penicillin/streptomycin. Cells were incubated in a humidified atmosphere of 5% CO_2_ at 37°C and passaged with 0.125% trypsin. Then, the MDA-MB-231/HER2 breast cancer cells were harvested, and 5 × 10^6^ cells were injected in the mammary fat pad of a female BALB/c nude mouse (OrientBio, Sungnam, Korea). A 30-gauge needle was used for injection. The injection volume was 0.1 mL containing tumor cells and medium/matrigel complex with 50% dilution. Tumor growth was evaluated for 14 days, and an *in vivo* tumor imaging was performed when the largest tumor diameter reached 1–1.5 cm.

### Validation of *in vivo* specific targeting by intraoperative multiplexed F-SERS dots

For an intraoperative multiplexed imaging by targeted F-SERS dots, the anti-HER2 antibody-conjugated F_AF610_-SERS_RITC _dots (anti-HER2-F_AF610_-SERS_RITC_ dots) and anti-EGFR antibody-conjugated F_AF610_-SERS_FITC_ dots (anti-EGFR-F_AF610_-SERS_FITC_ dots) were prepared. After making a minimal (*ca.* 10 mm) skin incision and exposing the tumors, two types of the tumor surface were studied: the outer surface, from which it is assumed to identify the tumor on their surface, and the cut surface, from which it is assumed to find tumors at the resection margins. For the outer surface study, the F-SERS dots were sprayed on the tumor, whereas the F-SERS dots were sprayed after a 5-mm cutting for the cut surface study. To validate the specific targeting ability of the F-SERS dots, the tumor site was pre-treated with blocking antibodies (monoclonal antibodies against HER2 or EGFR). For either single target blocking, HER2 or EGFR, the tumor sites were treated with 1 mg of anti-HER2 or anti-EGFR antibodies (injection volume, 200 μL of 5 mg/mL antibodies in PBS). For the dual target blocking, a tumor site was treated with 1 mg mixture of the anti-HER2 and anti-EGFR antibodies. After 10 minutes, these tumor sites were washed with PBS, and 100 μg (volume = 20 μL, estimated concentration: 0.28–0.63 μg/mm^2^) of the targeting F-SERS dots (anti-HER2-F_AF610_-SERS_RITC_ dots and anti-EGFR-F_AF610_-SERS_FITC_ dots, concentration = 5 mg/ml, respectively) were sprayed on the surface of tumor sites using a micro-pipette (AxyPet^TM^ Single-channel pipettor, Axygen, USA). After 10 minutes incubation, the F-SERS dots-sprayed tumors were washed three times with PBS (200 μL at each time) to remove unbound F-SERS dots using a micropipette. Subsequently, real-time fluorescence and Raman spectra at the tumor sites were measured using the FRES instrument. The antibodies pre-treatment, F-SERS dots treatment, and FRES probe insertion were performed on the same surface of the tumor.

## Author Contributions

S.Y.J. and Y.-i.K. contributed equally to the work, developing and characterizing the fluorescence-Raman endoscopic system (FRES) incorporating the fluorescence-SERS nanoprobes (F-SERS dots), and performing *in vivo* molecular diagnosis in the orthotopic breast cancer model by utilizing this system. Y.-S.L., K.W.K., D.S.L. and D.H.J. have designed this research and supervised this project. G.S.K., H.M.K. and B.-H.J. contributed by assisting and advising in the development of the FRES incorporating the F-SERS dots. D.W.H., Y.-S.L. and H.W.Y. contributed by analyzing in the bio-medical data of *in vivo* breast cancer actively targeting experiment. M.G.C. and H.J.C. contributed by assisting in the preparing the F-SERS dots. K.O.J. and Y.-H.K. contributed by assisting in the preparing the orthotopic breast cancer model.

## Supplementary Material

Supplementary Information Supplementary Video S1

Supplementary Information Supplementary Information

## Figures and Tables

**Figure 1:**
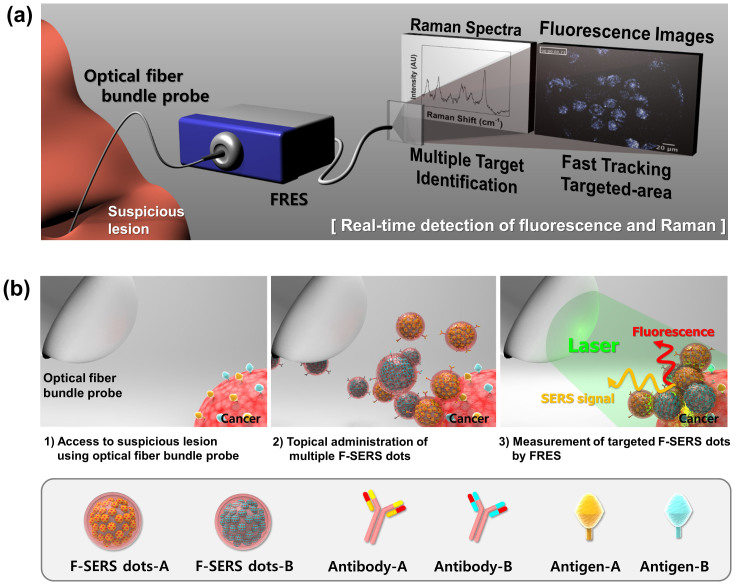
Schematic illustration of real-time multiplexed imaging using the fluorescence-Raman endoscopic system (FRES). (a) The mode of dual modal detection with fluorescence and Raman scattering. The real-time fluorescence imaging tracks the locations of the probe-targeted areas, and a concurrent SERS spectral analysis identifies the species of targets. (b) Illustration of the *in vivo* multiplexed molecular imaging procedure: First, access to a suspicious lesion *via* optical images; second, the spray-and-mix multiple F-SERS dots for topical administration; third, a multiplexed measurement of the targeted F-SERS dots with FRES.
