# Supplementary material for: Fluorescence-Raman Dual Modal Endoscopic System for Multiplexed Molecular Diagnostics
Source: Sci Rep. 2015 Mar 30;5:9455. doi: 10.1038/srep09455 (PMC4377550; doi:10.1038/srep09455)
Supplement: Supplementary Information [file srep09455-s2.doc]

**Fluorescence-Raman Dual Modal Endoscopic System
for Multiplexed Molecular Diagnostics**

(Supplementary Information)

Sinyoung Jeong,^a†^ Yong-il Kim,^b,c†^ Homan Kang,^d^ Gunsung Kim,^a^ Myeong Geun Cha,^a^ Hyejin Chang,^a^ Kyung Oh Jung,^e^ Young-Hwa Kim,^f^ Bong-Hyun Jun,^g^ Do Won Hwang,^b,h^ Yun-Sang Lee,^b^ Hyewon Youn,^b,h^ Yoon-Sik Lee,^d,i^ Keon Wook Kang, ^b^ Dong Soo Lee,^b,c*^and Dae Hong Jeong ^a,d*^

^a^ Department of Chemistry Education, Seoul National University, Seoul 151-744, Republic of Korea.

^b^ Department of Nuclear Medicine, College of Medicine, Seoul National University, Seoul 110-744, Republic of Korea

^c^ Department of Molecular Medicine and Biopharmaceutical Sciences, Graduate School of Convergence Science and Technology, Seoul National University, Seoul 151-747, Republic of Korea,

^d^ Interdisciplinary Program in Nano-Science and Technology, Seoul National University, Seoul 151-744, Republic of Korea

^e^ Laboratory of Molecular Imaging and Therapy, Cancer Research Institute, Seoul National University College of Medicine, Seoul 110-799, Republic of Korea

^f^ Division of High-Risk Pathogen Research, Center for Infectious Diseases and Prevention, Korea National Institute of Health, Seoul 110-799, Republic of Korea

^g^ Department of Bioscience and Biotechnology, Konkuk University, Seoul 143-701, Republic of Korea

^h^ Cancer Imaging Center, Seoul National University Cancer Hospital, Cancer Research Institute, Seoul National University College of Medicine, Seoul 110-799, Republic of Korea

^i^ School of Chemical and Biological Engineering, Seoul National University, Seoul 151-747, Republic of Korea

† These authors contributed equally to this work.

^*^ Corresponding authors: dsl@snu.ac.kr (D.S. Lee) and jeongdh@snu.ac.kr (D.H. Jeong)

KEYWORDS: Multiplexed Molecular Imaging, *in vivo* Diagnostics, Fluorescence, Surface-Enhanced Raman Scattering (SERS), Endoscopy


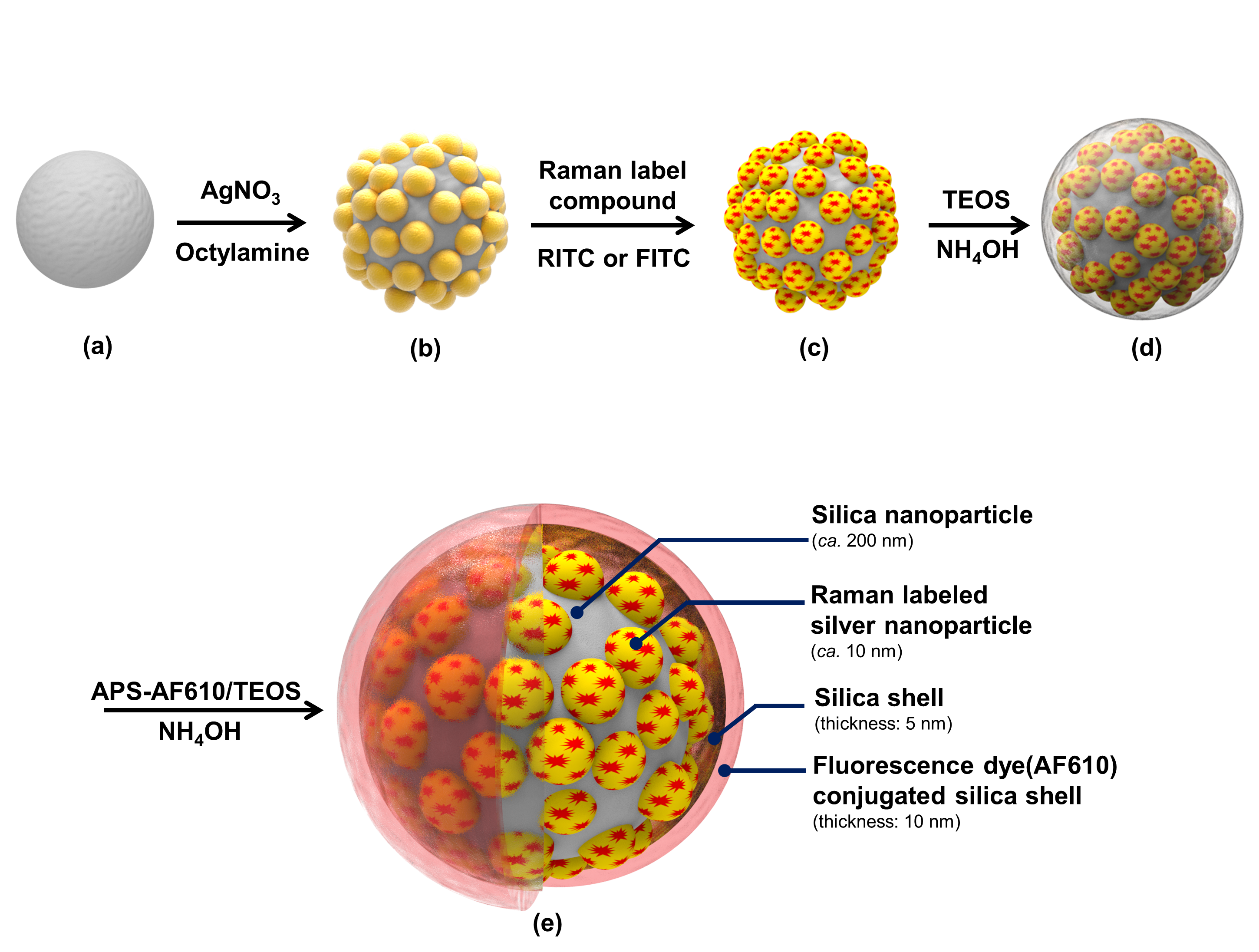


Figure S1. Schematic illustration of fabrication processes of fluorescence-SERS active nanoparticle (F-SERS dot). (a) Silica nanoparticle (NP), (b) silver-embedded silica NPs (Ag Si), (c) Raman-labeled Ag silica (SERS NPs) by RITC or FITC respectively, (d) silica shell-coated SERS NPs, and (e) fluorescence dye (AF610) conjugated silica shell coated SERS NPs (F-SERS dot).


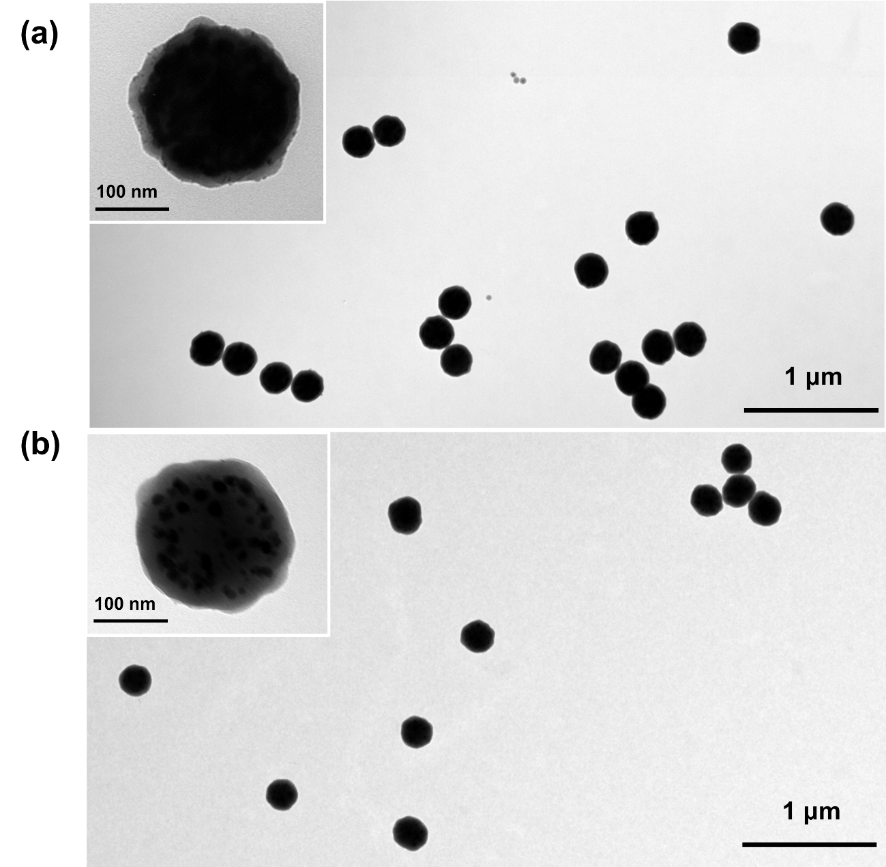


Figure S2. Transmission electron microscopy (TEM) images of (a) F_AF610_-SERS_RITC_ dots and (b) F_AF610_-SERS_FITC_ dots. The insets are highly magnified images of each F-SERS dot.

Figure S3. UV-Visible extinction spectra of AgNPs-embedded silica nanoparticles (Ag Si), F_AF610_-SERS_RITC_ dots, and F_AF610_-SERS_FITC_ dots


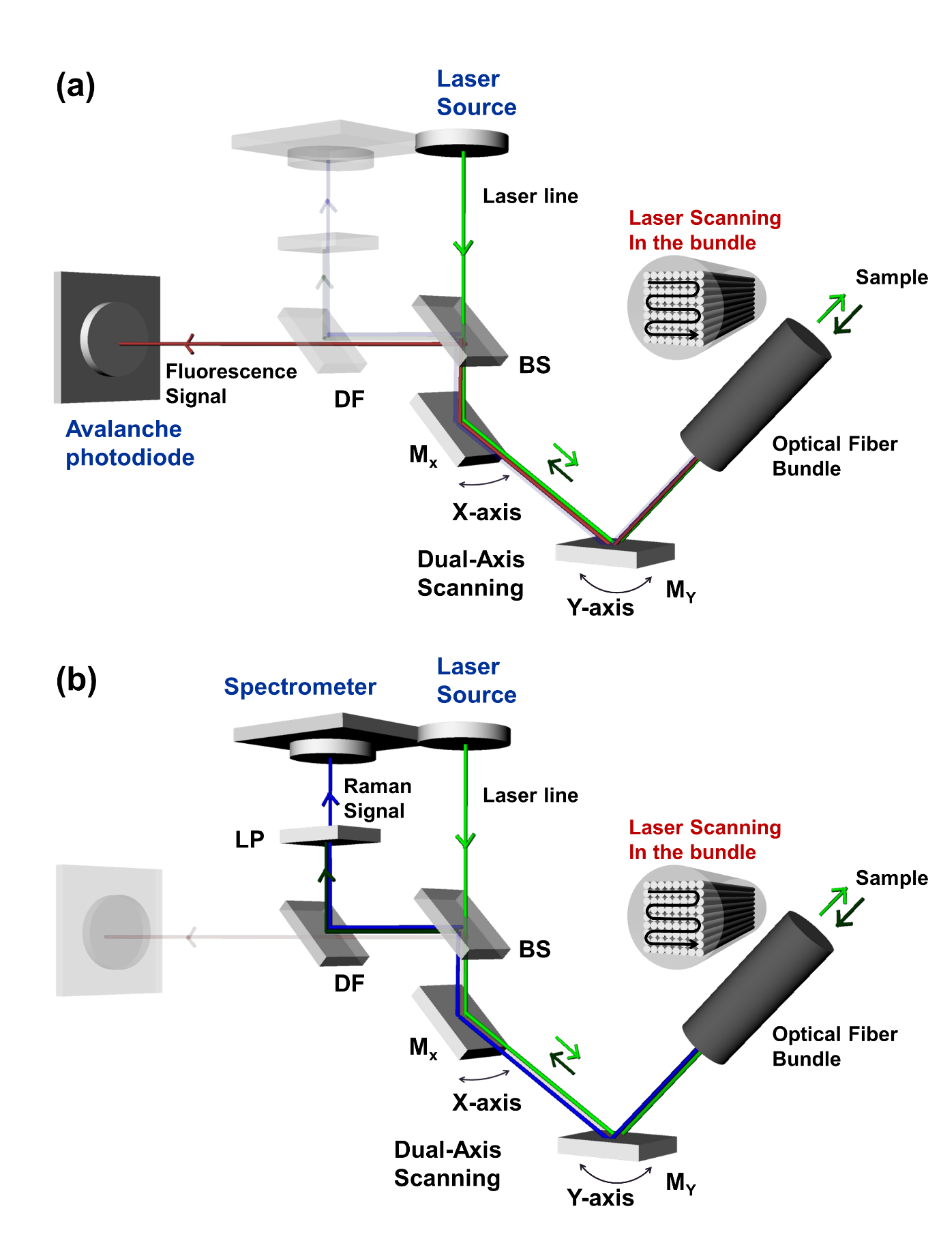


Figure S4. Schematic diagram of the optical beam path for (a) fluorescence imaging, and (b) SERS detecting. BS: Beam Splitter, M_X_: Oscillating mirror for X-axis, M_Y_: Oscillating mirror for Y-axis, DF: Dichroic Filter, and LP: Long-pass edge filter.


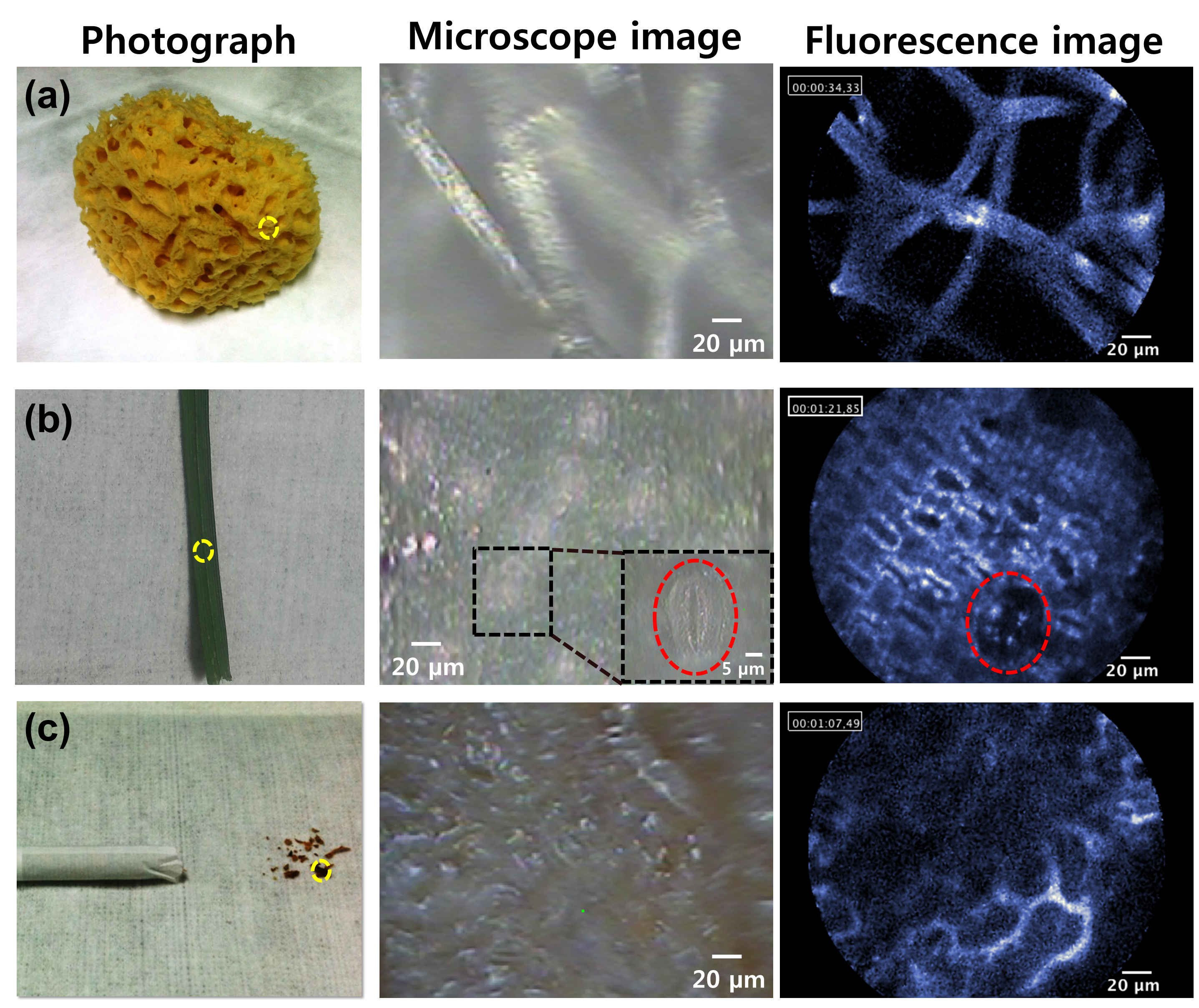


Figure S5. Demonstration of real-time fluorescence endomicroscopic imaging ability using the developed system. Macroscopic photographs, white light microscopic images, and fluorescence images of the samples: (a) Natural sponge, (b) leaf, and (c) cigarette. All fluorescence images were obtained by their auto-fluorescence signal with real-time (12 frames/s) and represented by a false color. The size of yellow circle represented in the macroscopic photograph is *ca.* 2 mm.


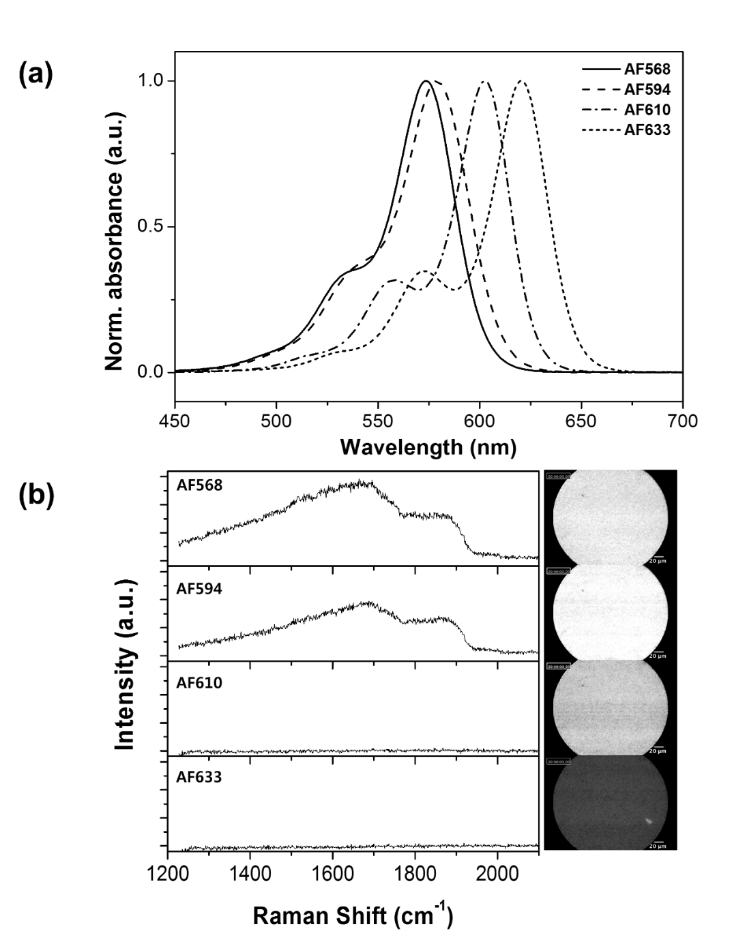


Figure S6. (a) The absorption spectra of candidate dyes for the F-SERS dots: AF568, AF594, AF610, and AF633. (b) The spectra in the Raman detection range from 1200 to 2100 cm^-1^ were obtained by photo-excitation with a 532-nm laser-line and measurement with the FRES. AF568 and AF594 reveal spectral overlap in this range by the fluorescence while the others not. Darker image for AF633 compared with relatively brighter image for AF610 indicates that AF633 is not effectively excited. Therefore, based on features in the spectra and the fluorescence images AF610 is chosen as the best fluorescence dye for the F-SERS dots among them, exhibiting little spectral overlap with the Raman detection range and proper the fluorescence excitation and emission. All Raman spectra were taken by FRES with laser power of 2.7 mW and acquisition time of 1s. Fluorescence images were obtained by FRES with real-time.


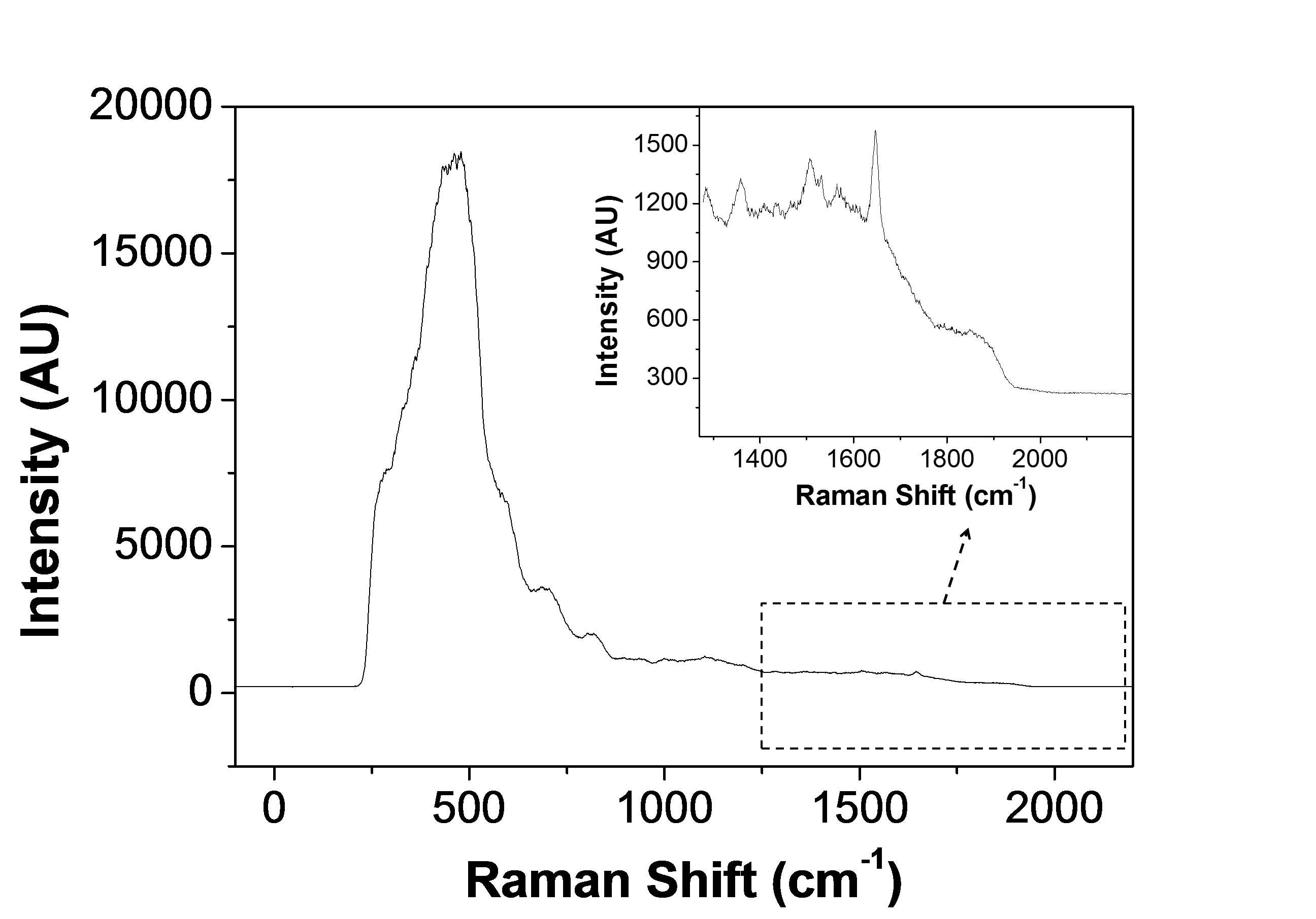


Figure S7. The Raman spectrum of the F_AF 610_-SERS_RITC_ dots through optical fiber bundle probe of the FRES. The strong noise pattern below 1250 cm^-1^ is caused by intrinsic optical noise by optical fiber itself. The inset is an enlarged spectrum of the SERS detectable range; 1250 ~ 2000 cm^-1^, which shows the distinctive SERS bands with background noise of moderate intensity.


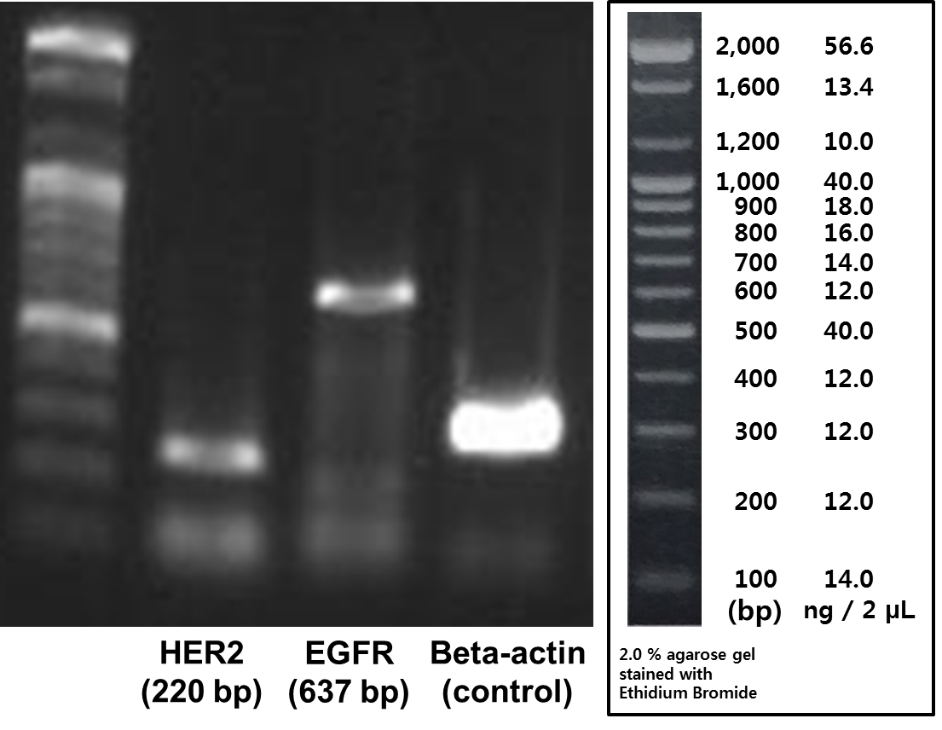


Figure S8. Reverse transcriptase-polymerase chain reaction (RT-PCR) for the HER2 and EGFR expression identification in the MDA-MB-231/HER2 breast cancer cell line.


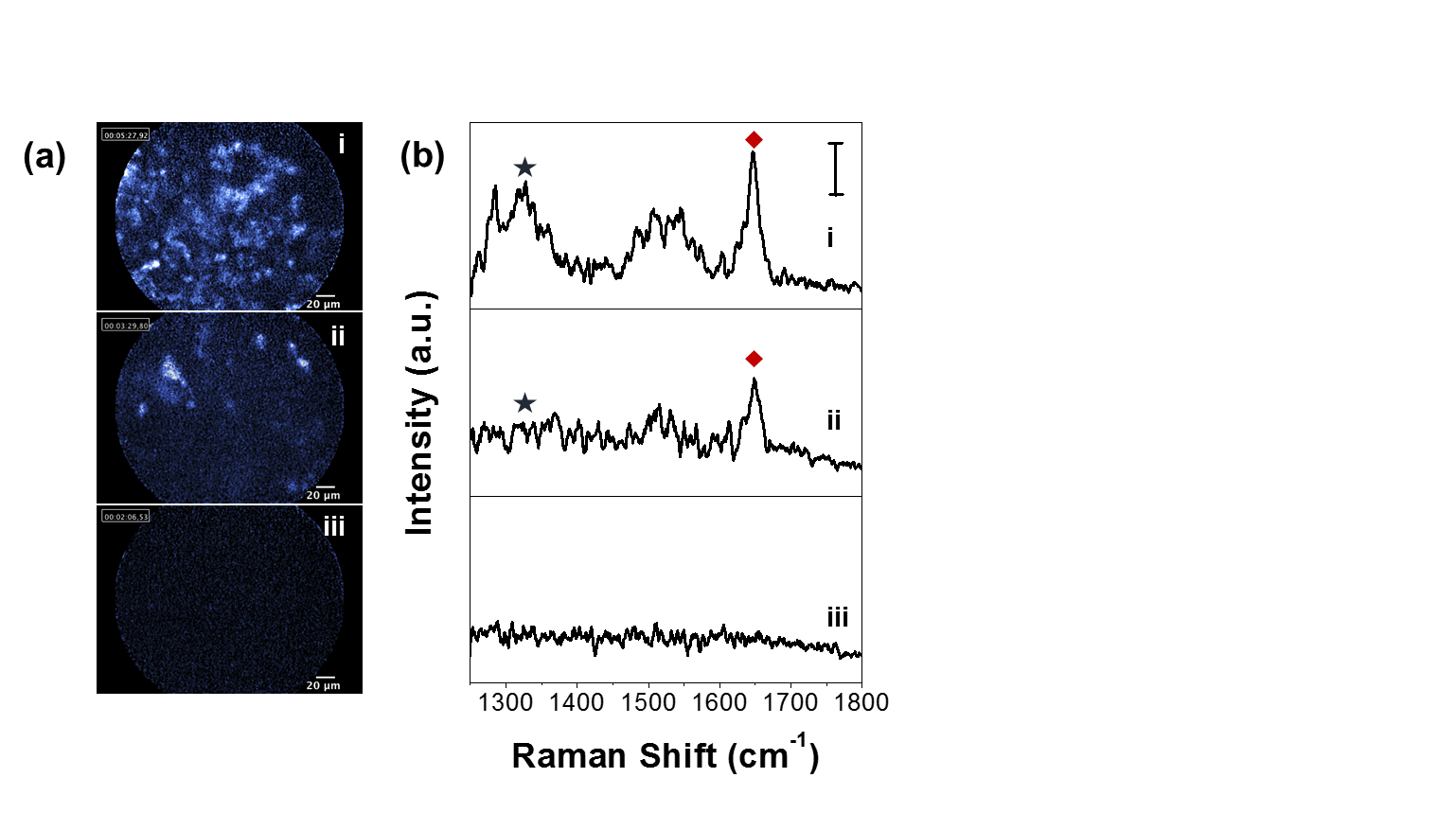


Figure S9. The representative (a) fluorescence images and (b) Raman spectra of each grades in whole results of the endoscopic multiplexed molecular diagnosis on the breast tumor xenografts. The grades of multiplexed detection are as follows: i) clearly detectable, ii) just-noticeably detectable, and iii) non-detectable. The scale bar in Raman spectra is 30 counts per second (cps). The labeled Raman bands in Raman spectra represents anti-EGFR antibody conjugated F_AF610_-SERS_FITC_ dots(★) and anti-HER2 antibody conjugated F_AF610_-SERS_RITC_ dots(◆), respectively.

Table S1. The grades of whole results of the endoscopic multiplexed molecular diagnosis on the outer and cut tumor surfaces of breast tumor xenografts in four mice. The results of multiplexed detection were graded according to the intensity of the representative Raman bands, 1324 cm^-1^ of FITC and 1648 cm^-1^ of RITC, as follows: a) clearly detectable, over the 30 counts per second (cps), which is three times of the noise level, b) just-noticeably detectable, from 10 to 30 cps, and c) non-detectable, from 0 to 10 cps. The noise level intensity was 10 cps, which was defined by the standard deviation of the background signals.

| **Multiplexed detection** | **Outer surface** | **Cut surface** | **Total** |
| --- | --- | --- | --- |
| Clearly detectable | 75.0% (3/4) | 100% (4/4) | 87.5% (7/8) |
| Just-noticeably detectable | 25.0% (1/4) | 0.0% (0/4) | 12.5% (1/8) |
| Non-detectable | 0.0% (0/4) | 0.0% (0/4) | 0.0% (0/8) |


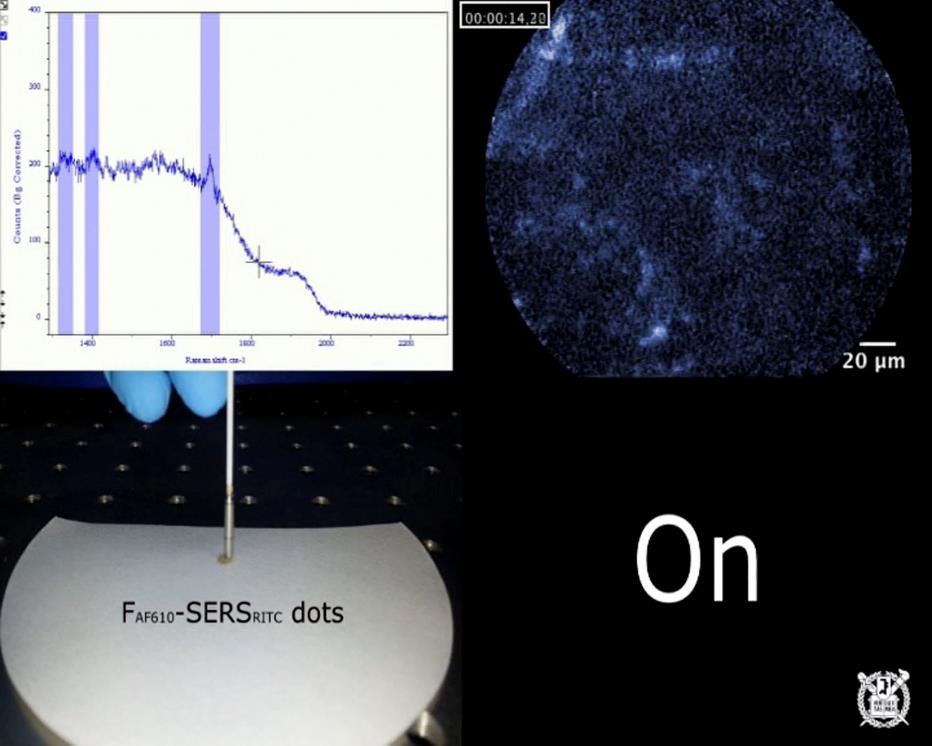


Video S1. Demonstration of the developed FRES system providing real-time fluorescence images and Raman spectra spontaneously and independently. All fluorescence images and Raman spectra were obtained using the FRES at a laser power of 2.7 mW. The Raman spectra were integrated for 500 ms.

Measurement of confocal microscope Raman system

As an additional characterization of fabricated F-SERS dots, Raman measurements were performed using a confocal microscope Raman system (LabRam 300, JY-Horiba, France) equipped with an optical microscope (BX41, Olympus, Japan). In this system, the Raman scattering signals are collected in a back-scattering geometry and detected by a spectrometer equipped with a thermo-electrically cooled CCD detector. The excitation source is the 532-nm line of a diode-pumped solid-state laser (CL532-100-S, CrystaLaser, Reno, Nevada). Focusing of the excitation laser lights and collection of Raman signals are accomplished by the same 100× objective lens (NA 0.90, Olympus, Tokyo, Japan). The strong Rayleigh-scattering light was eliminated by a long-pass edge filter. Raman spectra were acquired using 2.8-mW laser power, and a 1-s acquisition time. The fluorescence spectra were also obtained under the same excitation and acquisition condition.

Measurement of confocal laser scanning microscopy

Tumors were excised and fixed with 4% of paraformaldehyde, followed by staining nuclei using DAPI (4',6-diamidino-2-phenylindole). Confocal Laser Scanning Microscopy (CLSM; LSM 510 META, Carl Zeiss, Jena, Germany) was used for fluorescence signal detection. Excitation laser-lines for F-SERS dots and DAPI signals were 610 nm and 405 nm, respectively. Fluorescence signals of Alexa Fluor 610(F_AF610_) from F-SERS dots were detected and visualized. Data were analyzed with LSM Image Examiner software (Carl Zeiss).

Reverse transcriptase-polymerase chain reaction (RT-PCR) for the HER2 and EGFR expression identification in MDA-MB-231/HER2 breast cancer cell line

RNA was isolated by Trizol reagent (Invitrogen, Carlsbad, CA) in MDA-MB-231/HER2 cells, and cDNA was synthesized following the protocol (cDNA Synthesis Master Mix; GenDEPOT, Barker, TX). For PCR, the initial denaturation of 5 minutes at 94 °C, 30 amplification cycles of 30 s at 94 °C, 30 s at 60 °C, and 30 s at 70 °C, and 7 minutes extension at 72 °C was performed for HER2, EGFR and β-actin. The products of RT-PCR were resolved by 2% agarose gel electrophoresis. The DNA bands corresponded to HER2 and EGFR transcripts with 220 and 637 base pairs (bp), respectively.
